# Molecular mechanisms of perimenopausal cognitive impairment in a rat model: multi-omics integration reveals the Adgrl2-Camk2d-TRPV1 signaling axis mediating calcium homeostasis disruption and neuroinflammation

**DOI:** 10.3389/fnagi.2026.1862151

**Published:** 2026-07-14

**Authors:** Wen Wang, Shouhui Chai, Xianming Yin, Yanan Wei, Wenxue Sun, Yanqiu Wei, Juanjuan Shi

**Affiliations:** 1 Department of Gynecology, Tengzhou Central People's Hospital, Tengzhou, China; 2 Translational Pharmaceutical Laboratory, Jining No. 1 People’s Hospital Affiliated to Shandong First Medical University, Jining, China

**Keywords:** Adgr2, Ca^2+^ homeostasis, cognitive dysfunction, multi-omics, perimenopause, transient receptor potential channels

## Abstract

**Background:**

Perimenopause represents a pivotal transitional stage in female reproductive aging, often accompanied by neurological disorders such as cognitive decline. However, the underlying molecular mechanisms remain incompletely understood.

**Methods:**

In this study, a total of 18 eight-week-old female Sprague–Dawley (SD) rats were randomly divided into the control group and the perimenopausal syndrome (PMS) model group induced by bilateral ovariectomy (OVX). We systematically investigated the regulatory mechanisms of perimenopausal cognitive impairment by integrating behavioral assessments, histopathological examinations, biochemical and molecular validation, as well as multi-omics approaches, including transcriptomics, metabolomics, and ionomics.

**Results:**

The results showed that PMS rats displayed marked cognitive deficits alongside evident pathological alterations in hippocampal neurons. Differentially expressed genes were enriched in the inflammatory mediator regulation of transient receptor potential (TRP) channels pathway. Adhesion G protein-coupled receptor L2 (Adgrl2) and calcium/calmodulin-dependent protein kinase II delta (Camk2d) were synergistically upregulated, driving activation of the transient receptor potential vanilloid 1 (TRPV1) channel, which induced Ca^2+^ homeostasis imbalance and calcium overload. Adgrl2 was specifically highly expressed in microglia, accompanied by elevated levels of interleukin-6 (IL-6) and tumor necrosis factor-*α* (TNF-α), forming a vicious cycle of Ca^2+^-inflammation. Metabolomics analysis also revealed widespread metabolic disorders involving pathways such as amino acid and energy metabolism.

**Conclusion:**

The Adgrl2-Camk2d-TRPV1 signaling axis is a core driver pathway of perimenopausal cognitive impairment, exacerbating neuronal damage by mediating calcium overload, neuroinflammation, and metabolic disorders. This discovery provides a novel pathological mechanism for perimenopausal cognitive impairment, identifies Adgrl2 and TRPV1 as potential therapeutic targets, and designates Ca^2+^ as an ideal diagnostic biomarker, offering significant support for clinical diagnosis, treatment, and related theoretical research.

## 1 Introduction

Perimenopause is a critical midlife transitional stage leading to female reproductive senescence. Globally, over 850 million women aged 40–60 experience perimenopause, with approximately 88% developing related clinical symptoms (Brinton et al., 2015). Common manifestations include mood swings, sleep disorders, cognitive changes, vasomotor symptoms and sexual dysfunction (Nelson, 2008; Santoro, 2016; Sourouni et al., 2021; Chen et al., 2023). Among them, cognitive impairment is the most concerning clinical issue at this stage (Devi, 2018; Maki et al., 2018; Metcalf et al., 2023; Cunningham et al., 2025). Accumulating evidence indicates that perimenopause represents a well-recognized “vulnerable window” for female cognitive function. Approximately 50 to 70% of women experience impairments in verbal memory, information processing speed and attention (Metcalf et al., 2023). Barnett et al. (2025) revealed that normal-range systemic iron status was positively correlated with cognitive and brain function in perimenopausal women. Furthermore, multimodal imaging confirmed that this effect was not accompanied by abnormal elevation of cerebral iron deposition. Mosconi et al. (2025) confirmed via ^31^phosphorus magnetic resonance spectroscopy (31P-MRS) that perimenopausal subjective cognitive decline is closely associated with reduced mitochondrial ATP utilization efficiency in Alzheimer’s disease–susceptible brain regions. Notably, perimenopausal symptom severity is an early warning signal for long-term neurodegenerative risk, making mechanistic exploration critical for early intervention and risk reduction.

TRP channels are core cation channels responsible for regulating intracellular calcium homeostasis and mediating neuroinflammatory responses, participating in the synthesis of inflammatory mediators and cellular signal transduction (Zhang et al., 2021; Guo et al., 2025). Duitama et al. (2020) demonstrated that aberrant activation of TRP channels is involved in the pathological progression of cognitive impairment by modulating the crosstalk among Ca^2+^ homeostasis, oxidative stress, and inflammatory mediator production. As the primary mediator of extracellular Ca^2+^ influx, TRPV1 has been confirmed to be closely associated with microglial activation and hippocampal neuroinflammation, and serves as a core target for regulating neuronal calcium homeostasis and neuroinflammation (Chen et al., 2025; Tang et al., 2025). In microglia, TRPV1 agonists can induce calcium influx and exert anti-inflammatory effects by regulating core signaling pathways including NF-κB and MAPK (Chen et al., 2025). Meanwhile, TRPV1 channel-mediated calcium influx in the central nervous system has been identified as a key pathway for acupuncture-regulated neuroimmunomodulation (Tang et al., 2025).

Recent studies have found that Adgrl2 and Camk2d are key molecules involved in synaptic dysfunction and neuroinflammatory signal transduction. Adgrl2 is highly expressed in hippocampal neurons and is critical for excitatory synapse formation and neural circuit connectivity (Anderson et al., 2017; Lee et al., 2025). Camk2d, as a major subtype of the CAMK2 family, is a core downstream kinase that transduces calcium signals to regulate synaptic plasticity and neuronal survival (Rigter et al., 2024). Loss of Camk2d expression severely impairs neuronal migration and cortical development, and its dysregulation participates in neuroinflammatory responses via modulating calcium signaling pathways, resulting in neuronal damage (Rigter et al., 2024; Oladapo et al., 2025). In addition to neuroinflammation and calcium dyshomeostasis, cerebral metabolic dysregulation during perimenopause is also a key driver of cognitive impairment. Systemic energy metabolic dysregulation in perimenopause can propagate to the brain via the central-peripheral metabolic coupling mechanism, manifesting as cerebral glucose hypometabolism and reduced efficiency of mitochondrial ATP production, which further impairs cerebral energy supply and exacerbates neuronal vulnerability (Mosconi et al., 2021; Metcalf et al., 2023). However, most existing studies have investigated these pathological processes independently. To date, no study has systematically elucidated the interactive regulatory network among calcium dyshomeostasis, neuroinflammation, and metabolic dysregulation in perimenopausal cognitive impairment from an integrated multi-dimensional perspective.

To elucidate the intrinsic associations among the aforementioned pathological processes, we established an OVX-induced rat model of perimenopause and performed a systematic integrated analysis combining transcriptomics, metabolomics, and ionomics. This study aims to reveal the molecular mechanism underlying the synergistic regulation of calcium dyshomeostasis, neuroinflammation, and metabolic dysregulation in perimenopausal cognitive impairment, thereby providing a theoretical basis for the early diagnosis and targeted intervention of this disorder.

## 2 Methods

### 2.1 Reagents

SDS-PAGE Gel Preparation Kit (BL522A, Biosharp), Western Primary Antibody Dilution Buffer (BL506B, Biosharp), Western Protein Marker I (G2086, Servicebio), SDS-PAGE Sample Loading Buffer(5X) (BL502A, Biosharp), HRP-conjugated Goat Anti-Rabbit IgG(H + L) (SA00001-2, Proteintech), HRP-conjugated Goat Anti-Mouse IgG (SA00001-1, Proteintech), Transfer Buffer (10X) (PS109S, Epizyme), Tris-Glycine-SDS Electrophoresis Buffer (10X) (PS105S, Epizyme), Tris Buffered Saline with Tween-20 (BL315B, Biosharp), BCA Protein Assay Kit (PC0020, Solarbio), APS (ST005, Beyotime), Skim milk powder (1172GR500, BioFroxx), TEMED (ST728, Beyotime), ECL Ultrasensitive Chemiluminescent Substrate Kit (BL520A, Biosharp), RIPA Lysis Buffer (Strong) (BL504A, Biosharp), 4% Paraformaldehyde Fixative (BL539A, Biosharp), Nissl Staining Solution (Toluidine Blue)(BL999A, Biosharp), Eosin (E4009, Sigma), Hematoxylin (H9627, Sigma), TRPV1(66983-1-Ig, Proteintech), TRPV4(27,892-1-AP, Proteintech), TRPA1(19124-1-AP, Proteintech), Adgrl2(PA3648, Abmart), Camk2d(81014-1-RR, Proteintech), β-actin(20536-1-AP, Proteintech).

### 2.2 Construction of a PMS rat model

A total of 18 eight-week-old female SD rats weighing 250–270 g were purchased from Pengyue Laboratory Animal Breeding Co., LTD (Jinan, China). The animals were housed in the laboratory with a temperature of 23 ± 2 °C and a relative humidity of 40–50% under a 12 h light/dark cycle and had free access to food and water. After 7 days of adaptation, rats were randomly divided into two groups: Control and PMS groups (9 per group), and a PMS rat model was established using the bilateral OVX method. Briefly, rats were anesthetized via intraperitoneal injection of 1% sodium pentobarbital (40 mg/kg) before surgery, and then underwent bilateral oophorectomy, in which the posterior back was sterilized, incision was made, and the ovarian tubes (including the fat) under the ovaries were ligated with fine wires to remove the ovaries bilaterally, and then the horns of the uterus were put back into the abdominal cavity in the postoperative period. The validation of the model was conducted using the vaginal smear method, and the relevant detection methods, operating procedures, and experimental data have been reported in detail in our team’s previous research (Wei et al., 2024). All procedures involving animals were performed in line with the animal experimentation and welfare established by the Animal Ethics Committee of Jining First People’s Hospital (JNRM-2025-DW-019), all experimental procedures conformed to the ARRIVE guidelines.

### 2.3 Morris water maze

A total of 6 rats per group underwent behavioral testing. Morris water maze training commenced at week 7 postoperatively (Qu et al., 2013). The maze was a round tank (diameter 110 cm), filled with tepid water (22 ± 2 °C). The water was made opaque by the addition of charcoal-black ink. The maze was divided into four quadrants, with one containing a hidden platform (diameter, 10 cm). Rats were placed in the water in a quasi-random fashion and were allowed to search for the platform for 60 s and remain on the platform for 10 s. If the rats did not reach the platform in 60 s, they were placed onto the platform manually for 15 s. Each animal was trained four times per day, with 15–20 min between sessions, for 5 days. A video tracking system records the animal’s location, swimming distance and time. On the sixth day, the platform was dismantled, and the animals were put into the water starting from the other side of the original platform quadrant. The latency and number of times the animals crossed the original platform quadrant within 60 s were recorded (Lu et al., 2025).

### 2.4 Sample collection

After the behavioral experiments were completed, rats were deeply anesthetized by intraperitoneal injection of 1% sodium pentobarbital (40 mg/kg) and then euthanized by rapid cervical dislocation. This method conforms to the American Veterinary Medical Association Guidelines for the Euthanasia of Animals, ensuring immediate loss of consciousness and minimal animal suffering. Brain tissues and hippocampal tissues were rapidly dissected on ice. A portion of the tissues was immediately snap-frozen in liquid nitrogen and stored at −80 °C for subsequent transcriptomic, metabolomic, ionomic, Western blot, and ELISA analyses; another portion was fixed in 4% paraformaldehyde at 4 °C for 24 h, followed by paraffin embedding for histopathological staining and immunofluorescence staining.

### 2.5 H&E staining

Brain tissues (*n* = 3 per group) were fixed in 4% paraformaldehyde solution for 24 h. After fixation, the tissues were dehydrated through a graded ethanol series, cleared in xylene, and then embedded in paraffin blocks. The paraffin-embedded blocks were sectioned into 4-μm-thick coronal sections using a rotary microtome. The sections were mounted onto glass slides and baked at 60 °C for 1 h. The paraffin sections were dewaxed in xylene, rehydrated through a graded ethanol series, stained with hematoxylin for 5 min and eosin for 2 min, then dehydrated again, cleared in xylene, and finally sealed with neutral resin (Niu et al., 2024). The stained sections were observed and imaged under an Olympus BX53 microscope.

### 2.6 Nissl staining

Brain tissue slides (*n* = 3 per group) were washed with PBS three times. Then, they were subject to removal of paraffin via sequential immersion with 100% xylene for 15 min, gradient ethanol (100, 90, 70%) for 5 min, 2 min, and 2 min, respectively, and deionized water for 2 min. After that, slices were incubated with Nissl staining solution for 10 min at room temperature. Following two washes with deionized water, slides were dehydrated with 95% ethanol twice for 5 s each and then incubated with xylene twice for 5 min each, and finally sealed with neutral resin (Lu et al., 2025). The stained slides were observed and imaged under an Olympus BX53 microscope.

### 2.7 Transcriptome sequencing

Hippocampal tissues (*n* = 3 per group) were rapidly dissected on ice, and total RNA was extracted with TRIzol reagent according to the manufacturer’s instructions. RNA concentration and purity were detected using a Nanodrop 2000 spectrophotometer (NanoDrop Technologies, USA), and RNA integrity was assessed using an Agilent 5,300 Bioanalyser (Agilent, USA). Only high-quality RNA samples (OD260/280 = 1.8 ~ 2.2, OD260/230 ≥ 2.0, RIN ≥ 6.5, 28S:18S > 1.0) were used for subsequent library construction.

RNA-seq libraries were prepared using the Illumina® Stranded mRNA Prep Ligation kit according to the manufacturer’s instructions. Briefly, 1 μg of total RNA was subjected to poly(A) mRNA enrichment, fragmentation, random hexamer primed reverse transcription, second strand synthesis, end repair, A tailing, adapter ligation, and PCR amplification. The resulting libraries were sequenced on a NovaSeq X Plus platform with a paired end read length of 2 × 150 bp.

Raw paired end reads were trimmed and quality controlled using fastp with default parameters. Clean reads were aligned to the reference genome using HISAT2. Gene abundances were quantified using RSEM, and the expression level of each transcript was calculated according to the transcripts per million (TPM) method. Differential expression analysis was performed using DESeq2 with FDR < 0.05 and |log₂FC| >1 as the threshold for significantly differentially expressed genes(DEGs). Kyoto Encyclopedia of Genes and Genomes (KEGG) carried out by KOBAS was used for enrichment analysis.

### 2.8 Western blot

The brain tissue (*n* = 3 per group) was lysed with RIPA buffer. Protein concentration was determined using a BCA kit. The protein samples were mixed with loading buffer, then boiled at 98 °C for 5 min. Different samples containing equal amounts of protein were separated by SDS-PAGE and transferred to PVDF membranes. After blocking with 5% skim milk at room temperature for 1 h, the membranes were incubated overnight at 4 °C with primary antibodies (TRPV1, TRPV4, TRPA1, Adgrl2, Camk2d, and β-actin) in TBS-T. The next day, after washing the membranes three times, they were incubated with secondary antibodies for 1 h. The bands were visualized using an ECL chemiluminescence kit. The grayscale ratio was calculated using ImageJ software.

### 2.9 Ionomics

After weighing the hippocampal tissue samples (*n* = 6 per group), they were placed in a constant volume tube that had been cleaned and dried with deionized water, 20% nitric acid, and deionized water in sequence. An appropriate amount of concentrated nitric acid was added, and after covering, it was heated on a heating plate at 130 °C for about 2 h until the reddish-brown smoke dissipated and the solution became transparent, then heating was stopped. After cooling, it was diluted to a final volume of 25 mL with ultra-pure water. The samples were analyzed using the Nex ION 2200 Inductively Coupled Plasma Mass Spectrometry (PerkinElmer, Shelton, CT, USA). This method effectively eliminates multi-atomic ion interference through collision and reaction modes, achieving high sensitivity and high selectivity quantification of various elements. The quantified ions in this study include calcium ions (Ca^2+^), zinc ions (Zn^2+^), magnesium (Mg^2+^), barium ions (Ba^2+^), lead ions (Pb ^2+^), etc. The quantification of element content in samples was calculated using the formula:


$$\;X=\frac{C\times V_{2}}{V_{1}}$$

*X* represents the concentration of elements in the sample, expressed in nanograms per milliliter (ng/mL).
*C* detects the concentration of the detected elements, expressed in micrograms per liter (ug/L).
*V_1_* is the sample size, expressed in milliliters (mL).
*V_2_* refers to the volume of the sample in milliliters (mL).

To assess the precision of measurements, the relative deviation (RD) between two parallel samples was calculated using the formula:


$$\;\mathrm{RD}=\frac{|X_{1-}X_{2}|}{\bar{X}}$$

*RD* is relative deviation.
*X_1_* and *X_2_* are the element concentrations in two parallel samples (ng/mL).
$\bar{X}$ represents the average concentration of the two parallel measurements.

The relative standard deviation (RSD) was determined using the formula:


$$\;\mathrm{RSD}=\frac{\mathrm{SD}}{\bar{X}}\times 100\%$$

*RSD* is the relative standard deviation.

*SD* represents the standard deviation of the measurements, and $\bar{X}$ is the mean concentration.

To ensure data reliability, the linear correlation coefficient of the standard curve must be greater than 0.995. In addition, the RD between two independent measurements conducted under repeatability conditions should not exceed 20%. Furthermore, the RSD of these independent measurements must be less than 10%. The screening criteria for differential ions are set as FC > 1 or<1, and *p* < 0.05.

### 2.10 Immunofluorescence

The paraffin-embedded brain tissue sections (*n* = 3 per group) were dewaxed, subjected to antigen retrieval, permeabilized with 0.1% Triton X-100, and blocked with 5% BSA at room temperature for 2 h. They were then incubated overnight at 4 °C with primary antibodies (TRPV1, Iba-1, and Adgrl2), washed three times with PBS solution (each wash for 5 min), and incubated with corresponding fluorescence-labeled secondary antibodies at room temperature. DAPI was used for nuclear staining. After washing, the sections were mounted. The sections were imaged using a fluorescence microscope, and images were collected to observe the staining results. All images were acquired using a panoramic scanner (3DHISTECH Panoramic Scan). Fluorescence intensity was calculated using ImageJ software.

### 2.11 Enzyme-linked immunosorbent assay (ELISA)

Place the brain tissue (*n* = 3 per group) in pre-cooled RIPA lysis buffer (containing protease inhibitors), use a tissue sonicator in an ice bath to homogenize, then centrifuge at 12,000 × g for 15 min at 4 °C, and collect the supernatant. The protein concentration is determined using the BCA method. According to the manufacturer’s instructions, measure the cytokine concentrations using ELISA kits for IL-6 and TNF-*α*.

### 2.12 Metabolome

Hippocampal tissue samples (50 mg each) were added to 2 mL centrifuge tubes with a 6 mm grinding bead. Metabolites were extracted using 400 μL of extraction solution (methanol: water = 4:1, v/v) containing 0.02 mg/mL of internal standard (L-2-chlorophenylalanine). Samples were ground using a Wonbio-96c frozen tissue grinder for 6 min (−10 °C, 50 Hz), followed by low temperature ultrasonic extraction for 30 min (5 °C, 40 kHz). The samples were left at −20 °C for 30 min, then centrifuged at 13,000 g for 15 min (4 °C). The supernatant was transferred to injection vials for LC MS/MS analysis. A pooled quality control (QC) sample was prepared by mixing equal volumes of all samples and was injected at regular intervals to monitor the stability of the analysis.

Metabolomic detection was performed on the Thermo UHPLC-Exploris480 system at Shanghai Majorbio Bio-pharm Biotechnology Co., Ltd. (Shanghai, China). Chromatographic separation was performed on an ACQUITY HSS T3 column (100 mm × 2.1 mm i.d., 1.8 μm, Waters, USA), with the column temperature set at 40 °C, the flow rate set at 0.40 mL/min, and the injection volume set at 10 μL. The mobile phases consisted of solvent A (0.1% formic acid in water, acetonitrile = 95,5, v/v) and solvent B (0.1% formic acid in acetonitrile, isopropanol, water = 47.5:47.5:5, v/v). The elution gradient for positive ion mode was set as follows: 0–3 min, 0–20% B; 3–4.5 min, 20–35% B; 4.5–5 min, 35–100% B; 5–6.3 min, 100% B; 6.3–6.4 min, 100–0% B; 6.4–8 min, 0% B. The elution gradient for negative ion mode was set as follows: 0–1.5 min, 0–5% B; 1.5–2 min, 5–10% B; 2–4.5 min, 10–30% B; 4.5–5 min, 30–100% B; 5–6.3 min, 100% B; 6.3–6.4 min, 100–0% B; 6.4–8 min, 0% B.

Mass spectrometry was performed on a Thermo UHPLC-Exploris480 system equipped with an electrospray ionization source operating in both positive and negative modes. The conditions were: auxiliary gas heating temperature 400 °C; capillary temperature 350 °C; sheath gas flow rate 50 psi; auxiliary gas flow rate 15 psi; ion-spray voltage floating at 3,000 V (positive) and −2,800 V (negative); normalized collision energy rolling from 20 to 60 eV. Full MS resolution was 60,000 and MS/MS resolution was 15,000, with data-dependent acquisition over a mass range of 70–1,050 m/z.

Raw data were processed using Progenesis QI software for baseline filtering, peak identification, peak integration, retention time correction, and peak alignment. Metabolites were identified by searching against HMDB,^1^ Metlin,^2^ and an in-house database (Majorbio). QC reproducibility was assessed by excluding variables with a relative standard deviation (RSD) > 30% in QC samples. Data were normalized using the sum normalization method. Orthogonal partial least squares discriminant analysis (OPLS-DA) was performed using the R package “ropls” (version 1.6.2). Differentially expressed metabolites (DEMs) were defined as those with FC > 1 or <1, *p* < 0.05, and VIP > 1. Pathway enrichment analysis was carried out using the KEGG database.

### 2.13 Statistical analysis

All experiments were independently repeated at least 3 times, and data were expressed as mean ± standard error of the mean (mean ± SEM). Statistical analysis was performed using GraphPad Prism 9.5.1. For comparisons between two groups, the unpaired two-tailed Student’s t test was used. For data involving repeated measurements across multiple time points, a two-way repeated measures analysis of variance (ANOVA) followed by Bonferroni’s multiple comparison test was applied. *p* < 0.05 is considered statistically significant. Statistical significance, no significance (ns), *p value < 0.05, **p value < 0.01, ***p value < 0.001, ****p < 0.0001.

## 3 Result

### 3.1 Cognitive dysfunction and hippocampal histopathological changes in PMS rats

**Figure 1:**
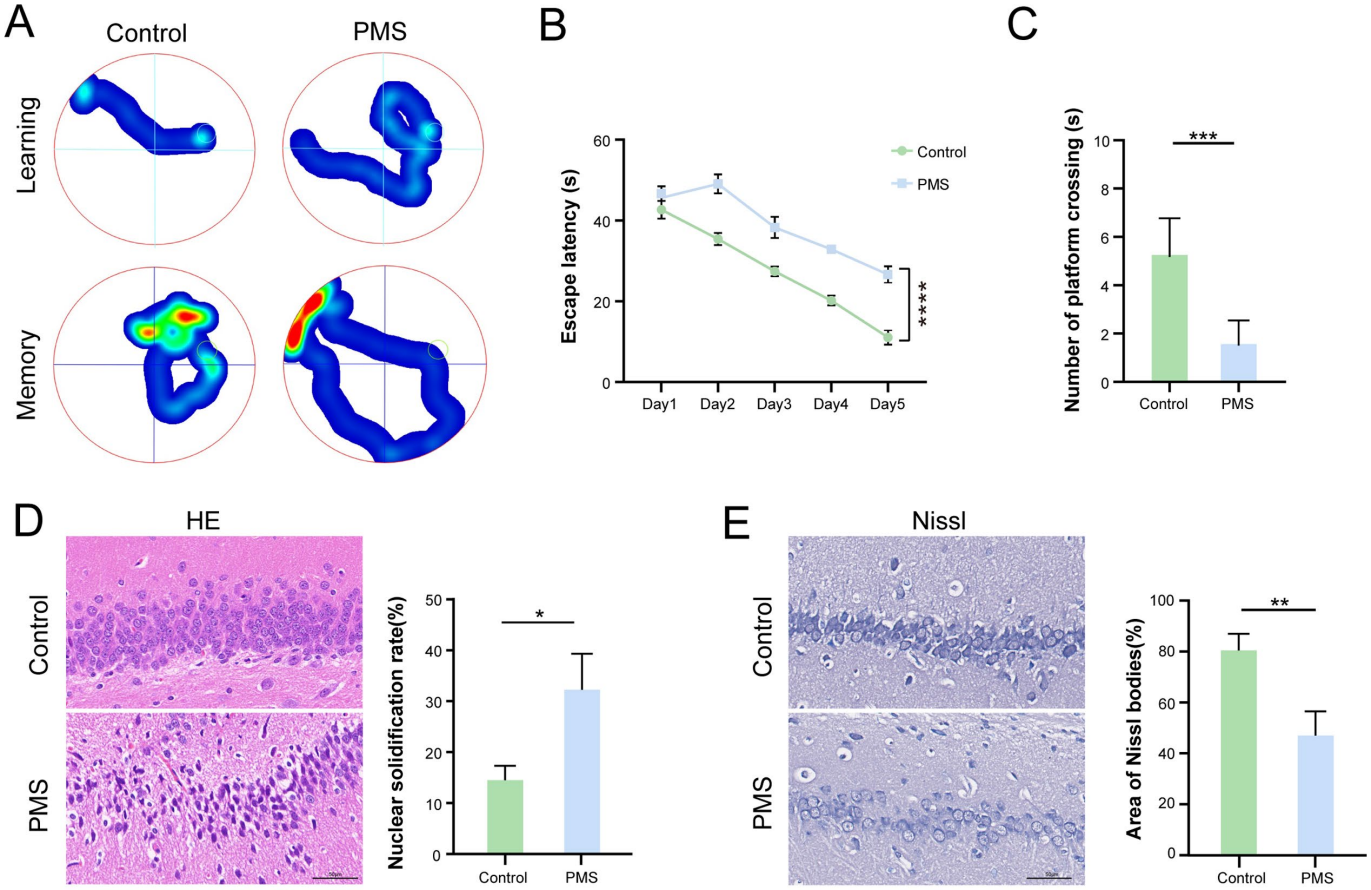
Behavioral and histopathological assessment of the hippocampus in the perimenopausal model rats. **(A)** Behavioral assessments of Control and PMS rats, PMS: perimenopausal group, n = 6 in each group. **(B,C)** Statistical analysis of escape latency and the number of platform crossings. **(D)** HE staining and statistical analysis of rat hippocampus. **(E)** Nissl staining and statistical analysis of rat hippocampus. **(D,E)** *n* = 3 in each group. *p < 0.05, **p < 0.01, ***p < 0.001, ****p < 0.0001.

To explore the impact of PMS on cognitive function and hippocampal pathology, this study developed a PMS rat model through bilateral OVX, followed by behavioral experiments and histological staining analyses. Behavioral analysis revealed that in the heatmap during the learning and memory phase (Figure 1A), compared to the control group, PMS group rats exhibited significantly reduced exploration behavior in the target quadrant. Further statistical analysis showed that PMS group rats had prolonged escape latency (Figure 1B) and fewer platform crossings (Figure 1C), indicating impaired spatial learning and memory abilities in PMS rats.

To clarify the histological basis of cognitive dysfunction in PMS rats, we performed H&E staining to observe neuronal morphology and Nissl staining to evaluate the degree of neuronal damage. H&E staining results (Figure 1D) showed that hippocampal neurons in the control group were neatly arranged with intact cell morphology, while those in the PMS group exhibited disordered arrangement and loose structure, and the proportion of neurons with nuclear pyknosis was increased by 121.72% compared with the control group. Nissl staining results (Figure 1E) further showed that compared with the control group, the number of Nissl bodies in hippocampal neurons of the PMS group was significantly reduced, their area proportion was decreased by 41.52%, and the staining intensity was significantly attenuated. These findings suggested that perimenopause may lead to impaired spatial learning and memory abilities, neuronal loss, and functional abnormalities in rats.

### 3.2 Differential gene expression analysis and functional enrichment results of the transcriptome

**Figure 2:**
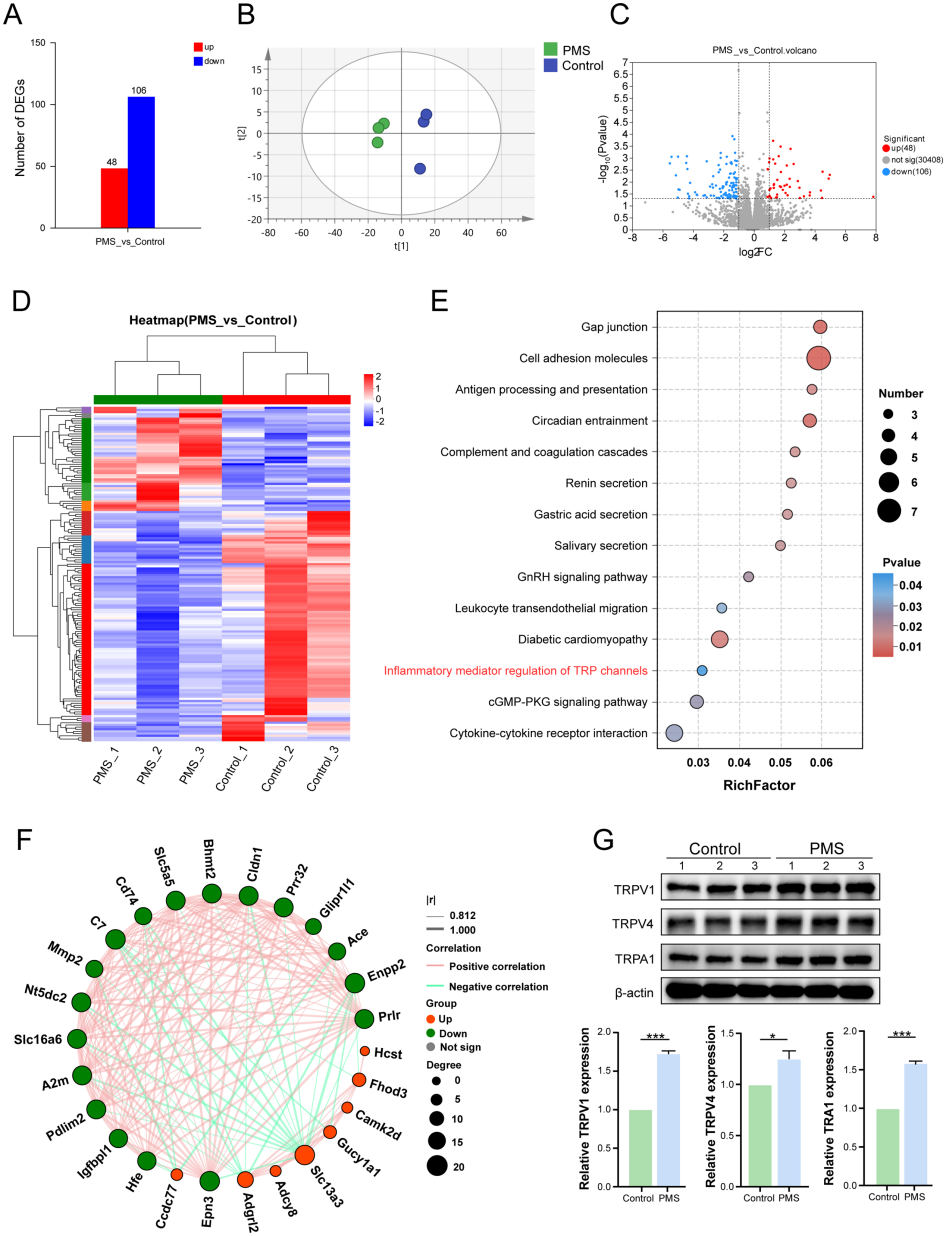
Transcriptome analysis. **(A)** Statistics of the number of differentially expressed genes in the transcriptome. **(B)** PCA plot. **(C)** Volcano plot. **(D)** Clustered heatmap. **(E)** KEGG enrichment analysis. **(F)** Correlation network analysis of differentially expressed genes. **(G)** WB detection of the expression levels of TRPV1, TRPV4, and TRPA1 and quantitative analysis. **(A–G)** *n* = 3 in each group. *p < 0.05, **p < 0.01, ***p < 0.001, ****p < 0.0001.

This study analyzed gene expression differences between samples through transcriptome sequencing. Figure 2A showed the statistical results of the number of DEGs identified by the transcriptome analysis. The principal component analysis (PCA) (Figure 2B) results indicated that the Control group and PMS group samples can be clearly distinguished at the transcriptome level, with good repeatability within groups and significant differences between groups. The volcano plot (Figure 2C) clearly displayed the distribution of DEGs, with 48 upregulated genes and 106 downregulated genes. The heatmap (Figure 2D) further validated the differences in expression patterns of DEGs between the two groups. Additionally, the differential gene correlation network analysis (Figure 2F) revealed the regulatory relationships of core DEGs. The results showed that the pro-inflammatory gene Camk2d and the neurodevelopment-related gene Adgrl2 were upregulated in the PMS group, while the anti-inflammatory gene Pdlim2 and the neuroprotective gene Ace were downregulated in the PMS group. The KEGG enrichment analysis (Figure 2E) results indicated that the DEGs were primarily enriched in signaling pathways such as Inflammatory mediator regulation of TRP channels and Cell adhesion molecules. To specifically validate the activation status of the TRP channel regulatory pathway, we selected three core functional proteins TRPV1, TRPV4, and TRPA1, within the TRP pathway for WB detection (Figure 2G). The result showed that the protein expression levels of TRPV1, TRPV4, and TRPA1 of the PMS group were significantly higher than those in the Control group, with increases of 71.85, 25.94, and 58.82%, respectively, directly supporting the findings of abnormal activation of Inflammatory mediator regulation of TRP channels in the transcriptome analysis.

### 3.3 Ionic group analysis and functional validation

**Figure 3:**
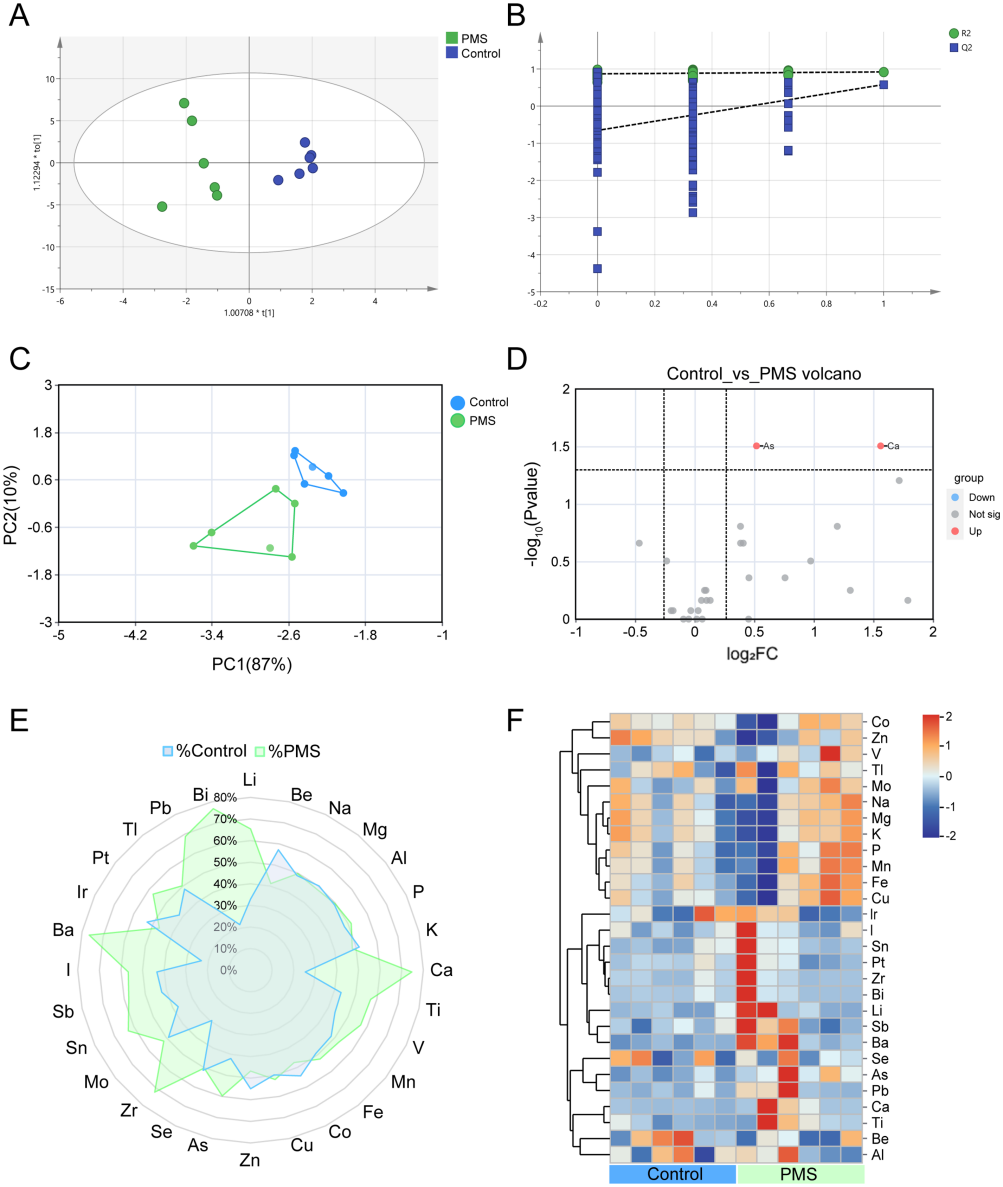
Ionomic analysis revealed changes in ion levels in the hippocampus of perimenopausal rats. **(A)** The OPLS-DA analysis revealed differences between the two groups. **(B)** Permutation test. **(C)** Ionome PCA plot revealed differences between the two groups. **(D)** Volcano plot of differential expressed ions. **(E)** Ionome radar chart. **(F)** Cluster heatmap. PMS, perimenopausal group. **(A–F)** *n* = 6 in each group.

To investigate alterations in ionic homeostasis in PMS rats, comprehensive ionomics profiling was conducted on hippocampus samples from both the control group and the PMS group. The orthogonal partial least squares discriminant analysis (OPLS-DA) model (Figure 3A) effectively distinguished the two groups of samples, demonstrating clear separation in their ionic profiles. And the permutation test (Figure 3B) verified the good stability of the model. The PCA results (Figure 3C) further showed significant separation in ionic composition between the two groups. The differential ion volcano plot (Figure 3D) visually presented the significantly different ions, among which ions such as Ca^2+^ and As^3+^ were upregulated. Additionally, the radar chart (Figure 3E) displayed the differential expression ratios of various ions (such as Pb^2+^, Bi^3+^, Ba^2+^, Ca^2+^, etc.) between the two groups. The clustering heatmap (Figure 3F) offered a detailed visualization of the expression patterns of multiple ions, clearly delineating the distinct ionic signatures of the Control and PMS groups. Collectively, these findings underscored the presence of significant ionic compositional differences between the Control and PMS rat groups.

**Figure 4:**
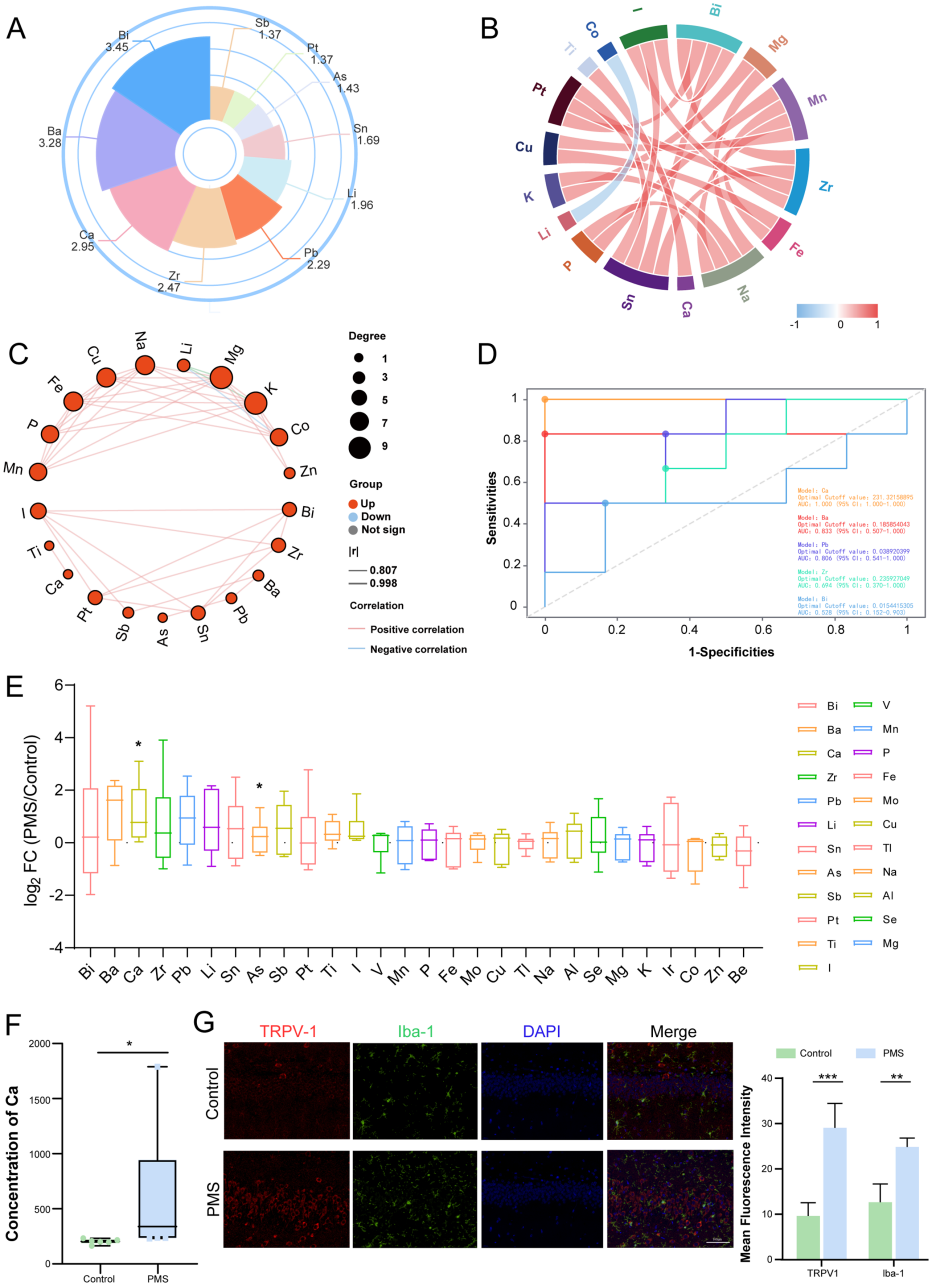
Analysis of ion group differential characteristics and functional validation. **(A)** Rose plot of ion FC values. **(B)** Chord diagram of ion correlations. The red connecting lines represent positive correlation, while the blue connecting lines represent negative correlation. **(C)** Differential correlation network among ions. **(D)** ROC analysis of differential expressed ions. **(E)** Boxplot of differentially expressed ions. **(F)** Boxplot of Ca^2+^. **(G)** Immunofluorescence detection of TRPV1 and Iba-1 expression in hippocampal tissue and quantitative analysis. PMS, perimenopausal group. **(A–F)** *n* = 6; **(G)** *n* = 3. *p < 0.05, **p < 0.01, ***p < 0.001, ****p < 0.0001.

To investigate the characteristics and functional associations of ion disorder in PMS rats, an in-depth analysis of the ionome was further conducted. The ion fold change (FC) rose plot (Figure 4A) visually presented the ranking of differential ions by their FC values, where Bi^3+^ exhibited the highest FC value (3.45), followed by Ba^2+^ (3.28) and Ca^2+^ (2.95). The volcano plot (Figure 3D) has clearly identified Ca^2+^ as a differential ion. These findings suggested that Ca^2+^ may play a central role in the ion homeostasis disturbances observed during the perimenopausal period. The chord diagram illustrating ion correlations (Figure 4B) and the differential correlation network (Figure 4C) collectively uncovered the intricate interaction relationships among various ions, with some ions exhibiting significant co-regulation patterns. The results of the receiver operating characteristic (ROC) analysis (Figure 4D) demonstrated that Ca^2+^ exhibited an area under the curve (AUC) value of 1.000, which was substantially higher than those of other ions, including Ba^2+^ (0.833) and Pb^2+^ (0.806). This finding indicated that Ca^2+^ has exceptional diagnostic discriminatory power and can be considered an optimal biomarker for detecting ion imbalances during the perimenopausal period. The boxplot of ion differential expression (Figure 4E) further quantified the distribution differences of various ions between the Control and PMS groups, with the difference in Ca^2+^ being particularly significant. Figure 4F focused on changes in Ca^2+^ concentration, showing that the Ca^2+^ content in the PMS group was significantly higher than that in the control group. Considering that TRPV1, as a core member of the TRP channel family, is a key channel mediating extracellular Ca^2+^ influx and that prior transcriptomic analysis had confirmed activation of the TRP pathway, we further detected the co-expression of TRPV1 and Iba-1 via immunofluorescence (Figure 4G). The results demonstrated that the fluorescence intensity of TRPV1 in the PMS group was increased by 206.85% and the fluorescence intensity of Iba-1 was increased by 93.8% compared with the control group, suggesting that upregulated TRPV1 expression in microglia under PMS conditions may mediate abnormal Ca^2+^ influx, thereby contributing to the activation of microglia. This provided direct cellular-level evidence for the association between Ca^2+^ homeostasis imbalance and neuropathological damage in PMS.

### 3.4 Association and functional validation of differentially expressed genes with calcium ions

**Figure 5:**
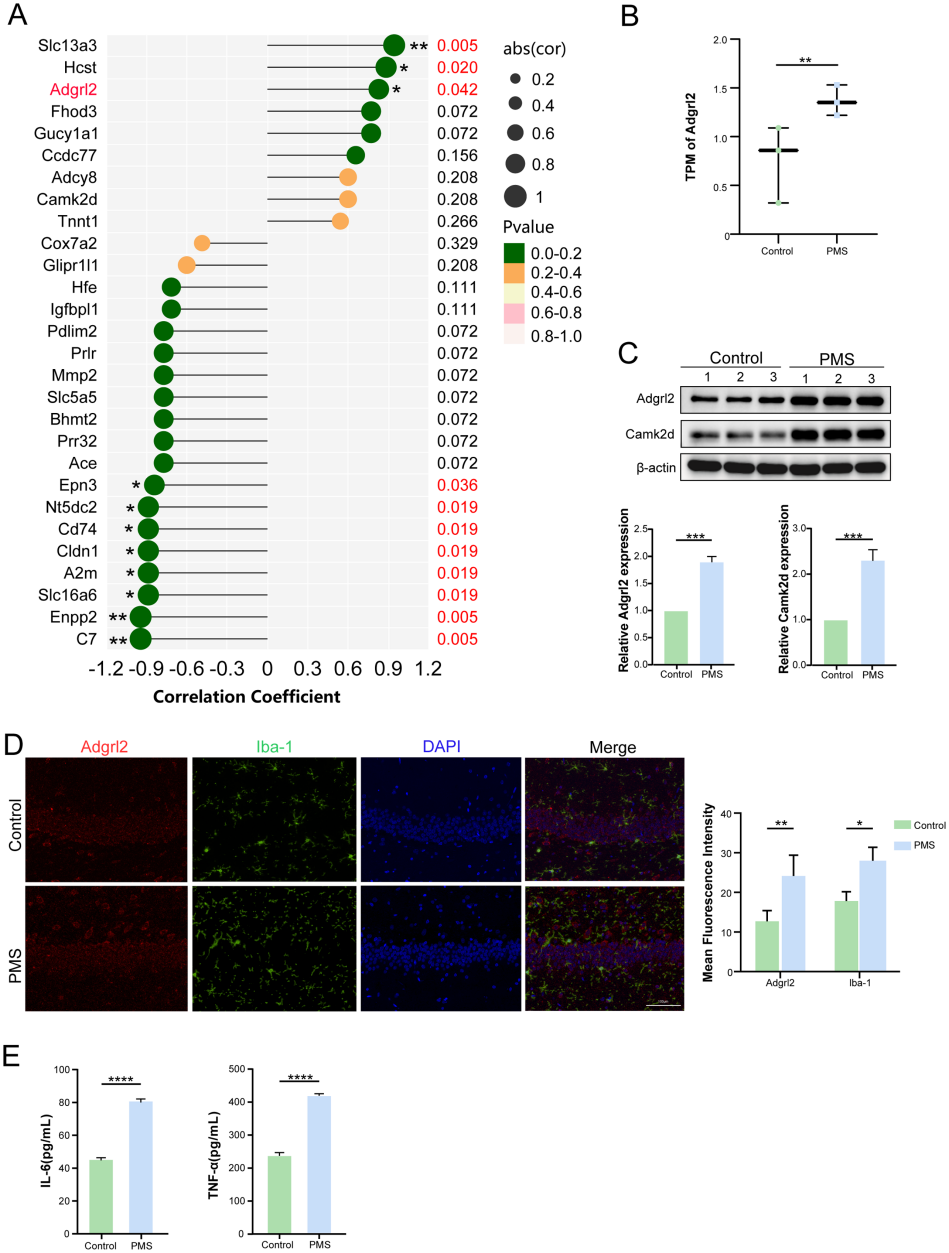
Analysis of the correlation between differentially expressed genes and Ca^2+^, as well as the expression of inflammatory factors. **(A)** Lollipop plot of the correlation between differential genes and Ca^2+^, Adgrl2 has a significant positive correlation with Ca^2+^. **(B)** The expression status of Adgrl2 in the transcriptome. **(C)** WB detection of the expression levels of Adgrl2 and Camk2d in the hippocampal tissue of Control and PMS rats, along with quantitative analysis. **(D)** Immunofluorescence detection of the expression level of Adgrl2 and its co-localization with Iba-1 in the hippocampal tissue of Control and PMS rats. **(E)** Elisa detection the expression levels of IL-6 and TNF-α in the Control and PMS group. PMS, perimenopausal group. **(B–E)** *n* = 3 in each group.*p < 0.05, **p < 0.01, ***p < 0.001, ****p < 0.0001.

To investigate the molecular mechanisms of Ca^2+^ homeostasis imbalance, we initially analyzed the correlation between DEGs and Ca^2+^. The correlation lollipop plot (Figure 5A) illustrated that numerous DEGs were significantly correlated with Ca^2+^, among which Adgrl2 exhibited a significant positive correlation with Ca^2+^. Meanwhile, transcriptome analysis revealed that the expression level of Adgrl2 in the PMS group was significantly higher than that in the control group (Figure 5B). To further clarify the protein-level expression characteristics and potential synergistic interactions of these Ca^2+^-related DEGs, we selected Camk2d, which also demonstrated a positive correlation with Ca^2+^, along with Adgrl2 for protein-level validation. Western blot results (Figure 5C) showed that the protein expression levels of Adgrl2 and Camk2d were significantly higher in the PMS group than in the control group, with up-regulation of 90.18 and 131.02%, respectively. This finding was consistent with the results of the transcriptome differential gene correlation analysis (Supplementary Figure 1). This indicated that these two genes are not only synergistically upregulated at the protein level but also exhibit a co-regulatory pattern in their transcriptional expression. Subsequently, the expression of Adgrl2 and Iba-1 was detected by immunofluorescence (Figure 5D). The results showed that the fluorescence signals of Adgrl2 and Iba-1 were weak in the control group, whereas both proteins exhibited significantly enhanced fluorescence intensity in the PMS group. Compared with the control group, the fluorescence intensities of Adgrl2 and Iba-1 in the PMS group were increased by 90.07 and 56.98%, respectively, suggesting a potential association between Adgrl2 and microglial activation. This finding implied that Adgrl2 may be involved in microglia-mediated neuropathological processes. The high expression of microglia was closely related to neuroinflammation, so we measured the levels of inflammatory factors in two groups of samples. The ELISA results (Figure 5E) showed that the expression levels of IL-6 and TNF-*α* in the PMS group were significantly higher than those in the control group, with increases of 78.05 and 74.33%, respectively, indicating a significant inflammatory response in PMS rats, which may be related to the abnormal regulation of Ca^2+^- related genes such as Adgrl2 and Camk2d.

### 3.5 Metabolomic analysis

**Figure 6:**
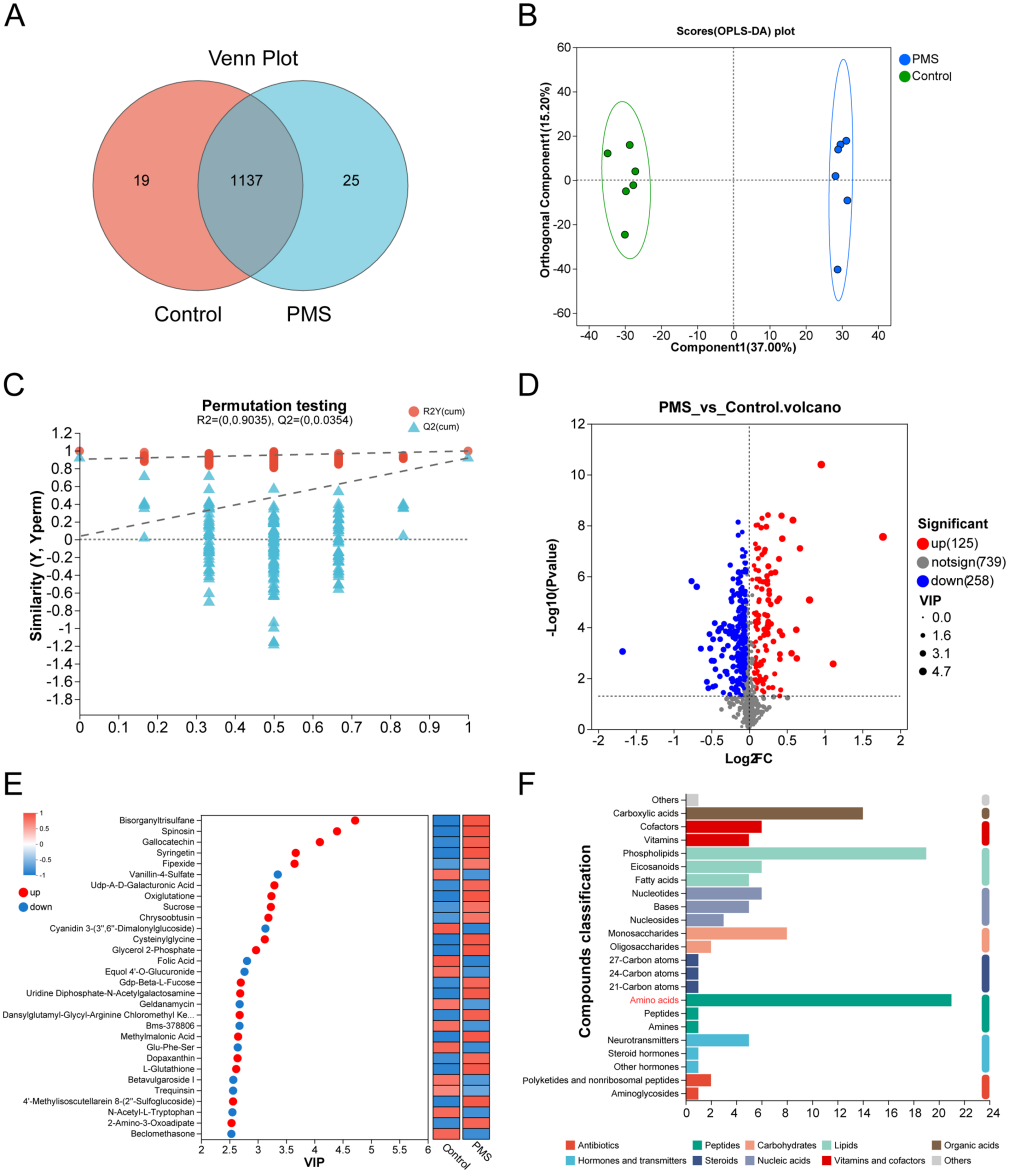
Metabolomic analysis reveals changes in metabolites in the hippocampus of perimenopausal rats. **(A)** Venn diagram of differential expressed metabolites between the Control and PMS groups. **(B)** The OPLS-DA analysis revealed differences between the control group and perimenopausal group. **(C)** Permutation test. **(D)** Volcano plot of differential expressed metabolites. **(E)** Combined VIP bubble plot and heatmap of differentially expressed metabolites. **(F)** Bar chart of metabolite classification. PMS, perimenopausal group. **(A–F)** *n* = 6 in each group.

**Figure 7:**
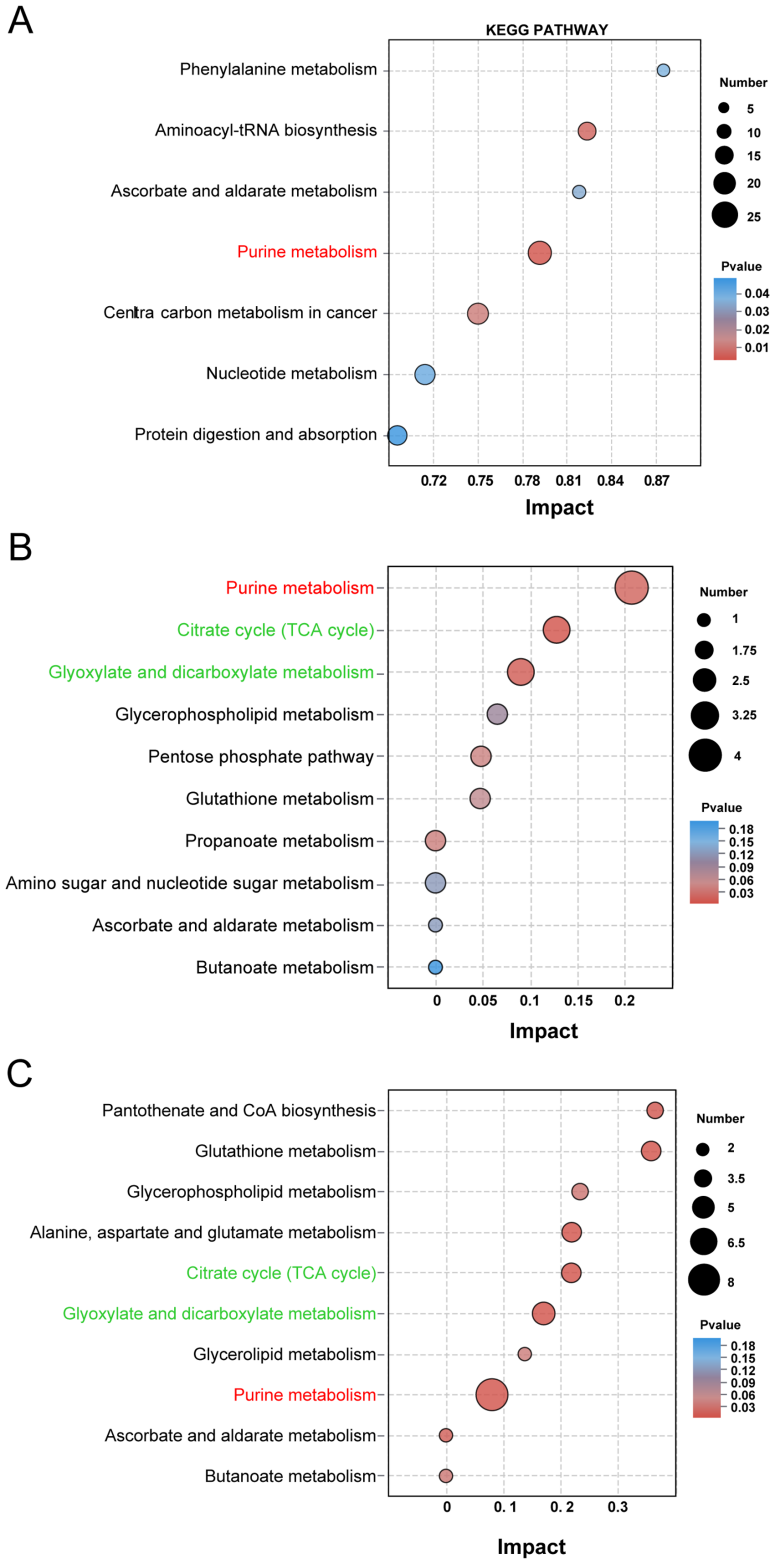
Metabolomics pathway enrichment analysis. **(A)** Bubble plot of KEGG enrichment analysis for differentially expressed metabolites. **(B)** Bubble plot of KEGG enrichment analysis for metabolites significantly correlated with Adgrl2. **(C)**, Bubble plot of KEGG enrichment analysis for metabolites significantly correlated with Ca^2+^.

To investigate the effects of PMS on metabolic homeostasis, metabolomic analysis was performed on two groups of samples. The Venn diagram (Figure 6A) showed a large number of DEMs between the PMS group and the control group. 1,137 metabolites were commonly altered in both groups, along with 19 unique DEMs exclusive to the control group and 25 unique DEMs exclusive to the PMS group. The OPLS-DA results (Figure 6B) revealed distinct clustering separation between the two groups. The reliability of the established model was further confirmed through a permutation test (Figure 6C). Additionally, the volcano plot of DEMs (Figure 6D) provided a visual representation of the distribution of these metabolites, where red dots indicated significantly upregulated metabolites and blue dots denoted significantly downregulated metabolites. The integrated VIP bubble plot and heatmap of DEMs (Figure 6E) highlighted key differential metabolites and their expression patterns, displaying those metabolites that contributed most to intergroup differences ranked by VIP values. The bar chart of metabolite classification (Figure 6F) showed that the DEMs were mainly concentrated in categories such as lipids, organic acids, amino acids, and their derivatives. Among them, the number of differential metabolites in the amino acids category was the highest. The KEGG enrichment analysis results showed that the DEMs were predominantly enriched in pathways such as Purine metabolism, Ascorbate and aldarate metabolism, and Nucleotide metabolism (Figure 7A). Metabolites significantly associated with Adgrl2 were mainly enriched in pathways including Purine metabolism, Citrate cycle (TCA cycle), and Glyoxylate and dicarboxylate metabolism (Figure 7B). Metabolites significantly associated with Ca^2+^ were primarily enriched in pathways such as Alanine, aspartate and glutamate metabolism, Citrate cycle (TCA cycle), Glyoxylate and dicarboxylate metabolism, and Purine metabolism (Figure 7C). These results collectively indicated that the PMS model induced significant changes in the composition of metabolites, involving multiple key pathways.

## 4 Discussion

**Figure 8:**
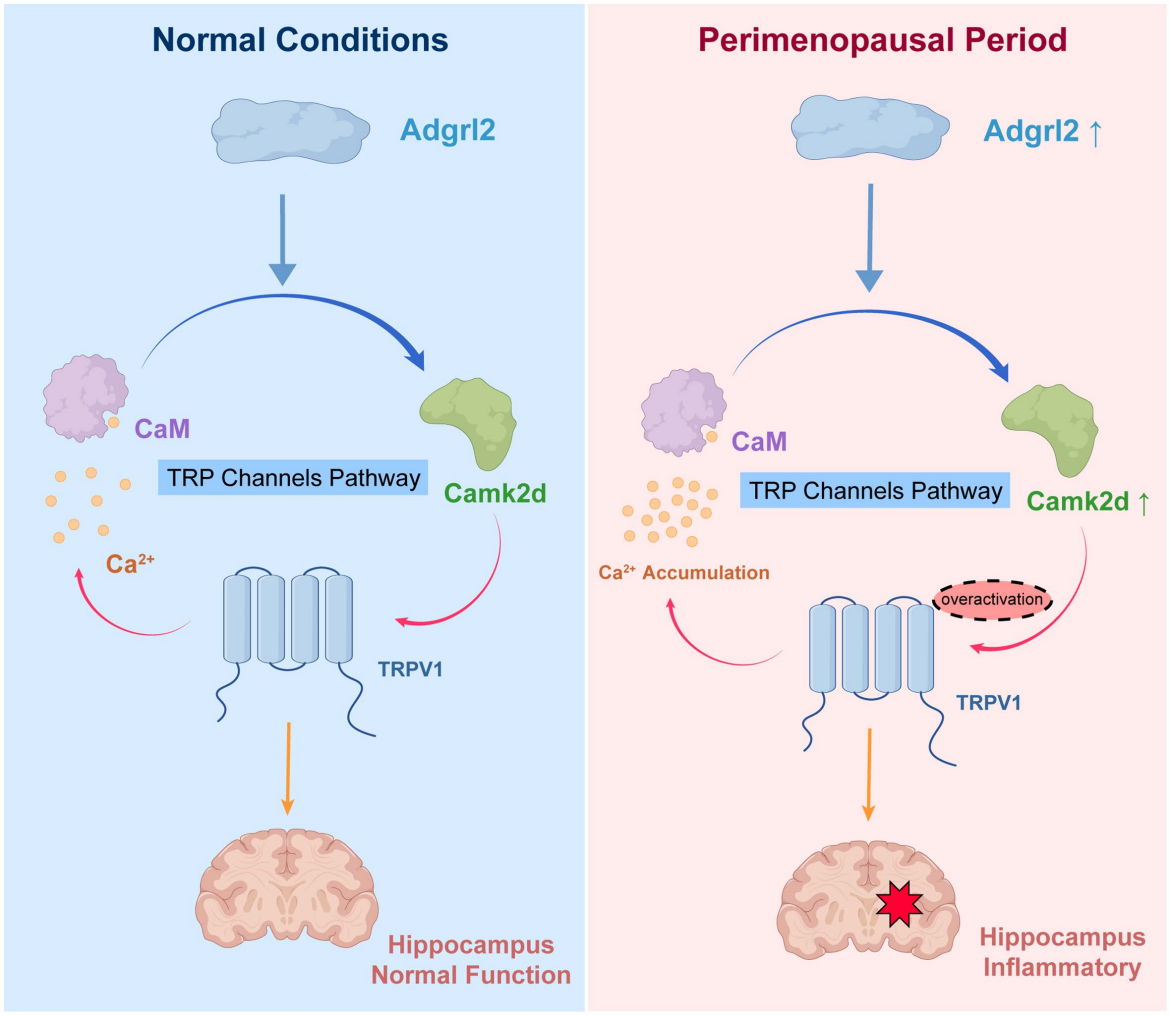
Mechanism diagram of Adgrl2 regulation of perimenopausal cognitive function. Adgrl2 and Ca^2+^ levels are upregulated in the hippocampal tissue of perimenopausal rats. Ca^2+^ binds to CAM to form a Ca^2+^-CAM complex; upregulation of Adgrl2 promotes the binding of this complex to CAMK2D, thereby activating CAMK2D. Upregulation of CAMK2D leads to excessive activation of TRPV1 channels, resulting in intracellular calcium overload, resulting in intracellular calcium overload and subsequent inflammation.

Clinical evidence indicates that women in the perimenopausal phase often display a range of cognitive impairments, including varying degrees of learning and memory deficits, as well as executive dysfunction. These manifestations can have a profound negative impact on their overall quality of life (Maki et al., 2021; Zhu et al., 2022; Choi et al., 2025). Nevertheless, the molecular mechanisms underlying these changes remain incompletely understood. This study investigated the core regulatory mechanisms of PMS in a rat model through multi-omics analysis and functional validation. The findings revealed that, under perimenopausal conditions, upregulated expression of Adgrl2 drives the synergistic activation of Camk2d, thereby enhancing the function of the TRPV1 channel. This leads to Ca^2+^ homeostasis imbalance and calcium overload, accompanied by disruptions in multiple metabolites and the onset of neuroinflammation, ultimately resulting in neuronal damage and cognitive decline (Figure 8).

Adgrl2, an adhesion G protein-coupled receptor, is closely associated with central nervous system dysfunction when aberrantly expressed in the hippocampus. Accumulating evidence has demonstrated that Adgrl2 regulates excitatory synapse formation and synaptic input efficiency, and is critical for spatial memory-related neural signal transduction (Anderson et al., 2017; Sando et al., 2019). In this study, we found that the expression level of Adgrl2 was significantly upregulated in the hippocampal tissues of PMS rats, and its expression level was significantly positively correlated with Ca^2+^ concentration, suggesting that the aberrant overexpression of Adgrl2 is closely associated with the development of hippocampal pathological damage during perimenopause. Meanwhile, we observed a highly synchronized upregulation trend in the expression of Adgrl2 and Camk2d. Under physiological conditions, the CaMKII family is a key regulator of synaptic plasticity, learning and memory formation, and neuronal survival (Lisman et al., 2012; Mohanan et al., 2022). Camk2d, as one of the core subtypes of this family, has an activity closely related to intracellular calcium concentration (Dauod and Taha, 2026). The synchronized aberrant expression of these two molecules may jointly participate in the regulation of hippocampal calcium homeostasis dysregulation and neurological impairment during perimenopause.

TRPV1 is a non-selective cation channel with high permeability to Ca^2+^, and aberrant expression and dysfunction of TRPV1 have been confirmed to be closely associated with calcium homeostasis dysregulation in central nervous system diseases (Saeed and Ma, 2025; Tang et al., 2025). In this study, TRPV1, TRPV4 and TRPA1 were all significantly upregulated, among which TRPV1 showed the most pronounced upregulation, indicating its key role in perimenopausal calcium homeostasis dysregulation. Previous studies have confirmed that sustained activation of TRPV1 channels mediates massive extracellular Ca^2+^ influx (Xiao et al., 2023; Zhang et al., 2025). When intracellular calcium concentration exceeds the buffering capacity of the calcium regulatory system and the tolerance level of cells, intracellular calcium overload will occur (Hu et al., 2024). Calcium overload is a classic core pathological event of neuronal injury, which can directly impair mitochondrial energy metabolism, trigger oxidative stress and apoptotic pathways, and lead to structural damage and functional abnormality of hippocampal neurons (Övey and Naziroğlu, 2015; Tang et al., 2025). The impaired spatial learning and memory ability, disorganized arrangement of hippocampal neurons, and reduced number of Nissl bodies observed in this study were highly consistent with the characteristics of neuronal pathological damage mediated by calcium overload.

More importantly, the aberrant expression of the above molecules and calcium homeostasis dysregulation are not independent pathological events, but have potential synergistic amplification effects with neuroinflammation and metabolic disorders. In this study, we found that the expression level of Adgrl2 was significantly positively correlated with the levels of hippocampal pro-inflammatory cytokines IL-6 and TNF-*α*, accompanied by evident microglial activation and elevated inflammatory factors in the hippocampus of PMS rats. A study by Wang et al. (2018) confirmed that pro-inflammatory cytokines such as TNF-α can enhance the functional activity of TRPV1 channels, suggesting that elevated inflammatory factors may further promote TRPV1-mediated Ca^2+^ influx, thereby exacerbating calcium homeostasis dysregulation and neuronal injury. There may be a mutually promoting pathological association between the two processes, which may also be an important reason for the progressive cognitive decline during perimenopause. Meanwhile, metabolomic analysis revealed significant metabolic profile dysregulation in the hippocampus of PMS rats, with metabolites mainly enriched in pathways including purine metabolism and tricarboxylic acid cycle. On the one hand, this metabolic dysregulation may be the combined consequence of calcium homeostasis dysregulation and neuroinflammation; on the other hand, it may further deteriorate the survival microenvironment of hippocampal neurons by impairing neuronal energy supply and normal physiological function, and collectively exacerbate cognitive impairment.

## 5 Conclusion

In summary, this study systematically reveals a multi-level pathological network driven by the Adgrl2-Camk2d-TRPV1 signaling axis in cognitive dysfunction during perimenopause. Aberrant activation of this signaling axis leads to Ca^2+^ homeostasis imbalance and calcium overload, thereby triggering microglia-mediated neuroinflammation and forming a self-reinforcing calcium-inflammation vicious cycle that directly damages neuronal structure. Meanwhile, the study also identified extensive metabolic remodeling associated with the perimenopausal state, involving key pathways such as amino acid and energy metabolism, which further exacerbated the disruption of the neural microenvironment. These findings not only elucidate a novel mechanism of cognitive decline during perimenopause from an integrated ion-molecule-inflammation-metabolism perspective but also provide potential targets like Adgrl2 and TRPV1 for clinical intervention. Additionally, they offer a theoretical basis for Ca^2+^-related molecular biomarkers in early disease diagnosis, enriching the molecular regulatory theory system of perimenopausal neuropathology.

## Data Availability

The transcriptome data has been uploaded to the SRA database of NCBI (PRJNA1490336). The data that support the findings of this study are available from the corresponding author upon reasonable request.

## Footnotes

1
http://www.hmdb.ca/

2
https://metlin.scripps.edu/

## Supplementary material

SUPPLEMENTARY FIGURE 1 Correlation heatmap among differentially expressed genes in the transcriptome.
