# Supplementary figures and images for: Molecular mechanisms of perimenopausal cognitive impairment in a rat model: multi-omics integration reveals the Adgrl2-Camk2d-TRPV1 signaling axis mediating calcium homeostasis disruption and neuroinflammation
Source: Front Aging Neurosci. 2026 Jul 14;18:1862151. doi: 10.3389/fnagi.2026.1862151 (PMC13408372; doi:10.3389/fnagi.2026.1862151)

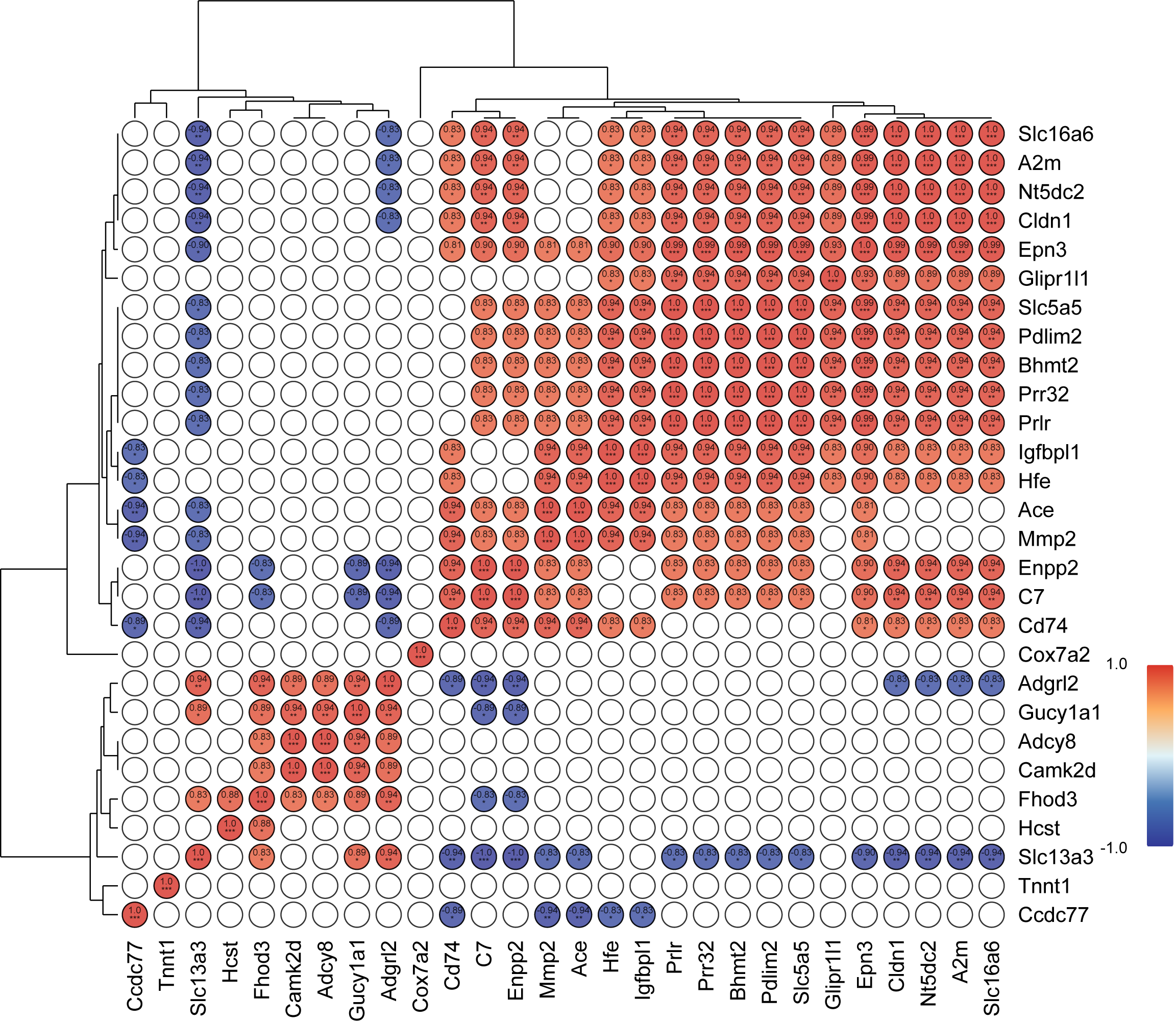

Supplement: SUPPLEMENTARY FIGURE 1 — Correlation heatmap among differentially expressed genes in the transcriptome. [file Image_1.TIF]
